# Rheological Relaxation of OSB Beams Reinforced with CFRP Composites

**DOI:** 10.3390/ma14247527

**Published:** 2021-12-08

**Authors:** Tomasz Socha, Krzysztof Kula, Arkadiusz Denisiewicz, Grzegorz Lesiuk, Wojciech Błażejewski

**Affiliations:** 1 Institute of Civil Engineering, Faculty of Civil Engineering, Architecture and Environmental Engineering, University of Zielona Góra, ul. Prof. Z. Szafrana 1, PL-65516 Zielona Góra, Poland; T.Socha@ib.uz.zgora.pl (T.S.); K.Kula@ib.uz.zgora.pl (K.K.); A.Denisiewicz@ib.uz.zgora.pl (A.D.); 2 Department of Mechanics, Materials Science and Biomedical Engineering, Faculty of Mechanical Engineering, Wroclaw University of Science and Technology, ul. Smoluchowskiego 25, PL-50370 Wrocław, Poland; Wojciech.Blazejewski@pwr.edu.pl

**Keywords:** CFRP, OSB, redistribution of stresses, relaxation, rheology, strengthening, wood, wood-based materials

## Abstract

An experimental and analytical approach to the relaxation problem of wood-based materials—OSB (Oriented Strand Boards—pressed wood-based composite panels) beams, including beams with CFRP (Carbon fiber reinforced polymer) tape composite reinforcement, is presented. It is a relevant engineering and scientific problem due to the fact that wood and wood-based materials, as well as composite reinforcements, are widely used in building constructions. Their rheological properties are very important and complicated to estimate. A 10 day long relaxation test of thick OSB beams without reinforcement and with CFRP tape was performed. A four-point bending test with five different bending levels was performed, during which the reduction of the loading force was measured. A five-parameter rheological model was used to describe the rheology of the beams. The equations of this model were calculated with the use of Laplace transform, whereas the values of the parameters were calculated based on the experimental relaxation curves. A high correlation between experimental and theoretical results was obtained. A beam reinforced with CFRP tape was treated as a system with a viscoelastic element (OSB) and an elastic element (CFRP), joined together without the possibility of slipping. The equations of the mathematical model were calculated based on the assumptions of the linear theory of viscoelasticity and the convolution integral. A good correlation between experimental and theoretical results was obtained. A significant redistribution of stresses was observed during the relaxation of the reinforced beam. The reinforced beams show a higher stiffness of approximately 63% and carry proportionally higher loads than unreinforced beams at the same deflection values.

## 1. Introduction

Wood is one of the oldest structural materials of our civilisation. However, its valuable properties make it still widely used, increasingly in the form of wood-based materials [1,2,3]. The benefits of wood and wood-based materials include their relatively low weight, relatively high compressive and tensile strength, and ease of processing and assembly. The ecological aspect is also not without significance. Wood and wood-based materials have a low thermal conductivity coefficient and are a renewable raw material. These advantages lead to using these materials in structures that were previously dominated by steel and reinforced concrete. Bridges are an excellent example of such structures. Wooden bridges were considered to be outdated, unsustainable, and expensive to maintain. The revival of such structures took place in the late 20th century due to the use of glued laminated timber, wood-based materials, and steel or fibrecomposite reinforcements [4,5].

Wood, despite its many advantages, also has disadvantages. It has many structural defects, and it is a heterogeneous and anisotropic material. Its properties depend on many factors such as humidity, temperature, and long-term loading [6]. The strength of wood decreases due to structural defects and/or inappropriate fibre orientation. Despite its high chemical resistance, it is vulnerable to various pests and fungi. These disadvantages can be eliminated by using glued laminated timber and wood-based materials. The properties of wood can be improved by reinforcing wooden structures with steel or composites. The fibrecomposite material deserves particular attention. The technological development of FRP composites leads to their wider use in the strengthening of wooden elements. They provide greater load-bearing capacity and stiffness of the elements. A similar coefficient of thermal expansion to wood allows both materials to be fully bonded. FRP layers are made of polymer matrix and longitudinal fibres: CFRP contains carbon fibres, GFRP—glass fibres, and AFRP—aramid fibres. Because of their many advantages, in particular the chemical resistance of carbon fibres to alkaline environments, high modulus of elasticity, and tensile strength of the composite, CFRP materials have found the widest application in the building industry. Research on composite-reinforced timber structures dates back to the 1980s, and many papers on the subject have been written since then. The most important and up-to-date review papers on this topic include the proceedings of the conference Innovative Wooden Structures and Bridges, Lahti 2001 [7,8,9]. Computational models for design purposes are presented in the paper by Blass and co-authors [10], and further models are contained in the articles by Kim et al. [11], Juvandes et al. [12], Glisovic et al. [13], Schober et al. [14], and Yang [15]. A very extensive review of computational models of beams strengthened with FRP strips was presented in the work of Nowak [16] and Łagoda [17]. The latter also focused on the strengthening of bridge structures with different materials. Additionally, Buell and Saadatmanesh presented the possibility of using FRP tapes for construction and strengthening of wooden bridges [18]. An extensive review of FRP fibres used for strengthening wooden structures was presented in work of André [19].

Epoxy resins are typically used to bond FRP tapes to the reinforced material. The epoxy resin molecule contains various functional groups, both polar and non-polar, which results in high adhesion of these resins to many materials. The high mechanical parameters of these resins make it possible to use them not only as an adhesive layer but also as an independent structural reinforcement [20,21,22,23,24,25].

It is crucial to know the distribution of strains and stresses in the reinforced element or structure and their distribution in time. The method of equivalent cross-section properties could be useful for estimating the stress distributions depending on time. In calculations, reinforcement is replaced by a section of wood or wood-based material with appropriately increased geometric dimensions. The increase depends on the elastic moduli ratio of the combined materials, as presented in Jasieńko [26], Masłowski and Spiżewska [27], and Mielczarek [28]. The model was used to analyse the behaviour of a segmental reinforcement of an I-beam with an OSB web and wood flanges in Socha et al. [29].

The American Standards, 30 US standard ICBO/Uniform Building Code 5100 [30] and US standard ICBO/Uniform Building Code 6046 [31], present algorithms for determining the load capacity and stiffness of glued laminated timber beams (GLULAM) with reinforcement in the tensile zone or in the tension and compression zone, with composite strips during manufacture. The load capacity of the reinforced section is determined assuming full plasticisation of the compressive zone. The timber load capacity is determined by exhausting the compression zone’s resistance and neglecting the wood’s contribution in the tensile zone. Tensile stresses are assumed to be carried by the FRP strip.

Wood and wood-based materials, as well as glues for fixing FRP strips, exhibit viscoelastic properties. This cannot be ignored in structural design, as the rheological deflection increments can exceed the initial elastic values several times over. In structures composed of several materials (e.g., wood reinforced with FRP), stress redistribution may occur due to the different rheological properties of the composite materials. It is essential to select an appropriate rheological model and identify its parameters.

Problems of OSB rheology are presented in works of Von Haas and Frühwald [32] and Sekino i Korai [33]. The obtained results indicate the rheological behaviour of the OSB is similar to that of wood. The authors did not propose any specific rheological model.

Building a proper rheological model for wood and wood-based materials is a challenging task. Wood and wood-based materials are susceptible to temperature and humidity changes and UV radiation, in addition to the previously noted wood defects. Rheological strain increments are also dependent on the cross-sectional dimensions of the component. These are anisotropic materials, which makes it additionally challenging to prepare a mathematical description of their behaviour.

Due to a strongly heterogeneous structure of wood and, in consequence, the impossibility of selecting samples with similar structure and properties for testing (the problem is particularly visible in the case of elements on a natural scale), the results of such tests are characterised by significant scatterings of values, even in the case of maintaining constant thermal and humidity conditions. An illustration of this fact may be the research results carried out by a team led by Fridley [34]. Deflections were measured in the middle of the span of supported pine girders with dimensions of 3.0 cm × 18.4 cm × 391.0 cm over 120 days. The elements were unloaded in the middle of this period. The initial elastic deflections ranged from 8.27 mm to 21.34 mm, and the rheological deflection increments (relative to the initial deflections) varied from 31 to 94%.

Considering the aforementioned information, the large number of wood rheology models used by many researchers is not surprising. The most commonly used models are: power-law models, three-parameter standard model, four-parameter Bürgers model, and models constructed on their basis supplemented with elements describing wood behaviour in variable heat and moisture conditions.

The power-law model was used by Clouser [35], Gerhards [36], Hoyle [37], and Fridley and Soltis [38,39,40], among others. The empirical power creep model exhibits several drawbacks. First, this model can only be used to describe creep at a constant load in time. Second, the constants appearing in its equations do not have a clear physical sense.

The models constructed from linear Hooke’s and Newton’s elements help to eliminate the disadvantages of the empirical power-law model. Initially, the three-parameter standard model and the four-parameter Bürgers model were used to describe wood rheology. Hoyle and Senft carried out work related to using these models, as did Suddarth and Fridley [39,40,41] and Brokans and Ozola [42]. The four-parameter model can be used to predict the long-term bending behaviour of beams. However, under varying climatic conditions, the behaviour of the tested specimens was simulated with significant discrepancies.

Toratti [43] proposed several ways to model the rheology of wood. One proposed model consists of seven Kelvin–Voigt bodies connected in series and an additional element to account for hydroexpansion.

According to Hanhijärvi [44], the rheological model consists of nine Maxwell bodies and one spring in parallel order. A component for the hygro-expansion is added to each parallel set of elements.

According to Becker [45], the rheological model consists of four Kelvin–Voigt bodies representing the ordinary creep and one Kelvin–Voigt-body representing the mechano-sorptive creep. Additional elements are added to consider the inelastic strains due to moisture variation and non-linear creep.

An extensive review of other models used to describe the rheology of wood is presented in the work of Holzer [46], Granello [47], and especially Schänzlin [48].

Rheological phenomena are also the main problem in composite and reinforced structures. Each of the materials used may exhibit different rheological properties, and their interactions may lead to a significant redistribution of stresses. Plevris and Triantafillou [49] carried out rheological tests on beams reinforced with CFRP strips. The authors used a five-parameter Bürgers–Fridley model to describe the rheology of the wood, whereas the CFRP tape used a power model. The Bürgers model was also used by Davids et al. [50] on the strengthening of glued laminated timber beams with glass fibre. A similar mathematical description was presented in the work of Yang et al. [51].

Jasieńko and Nowak [52,53] have conducted a comprehensive testing programme of reinforced timber beams and timber-reinforcing glue joints. Destructive and long-term tests were performed on wooden beams without and with reinforcement on a laboratory and natural scale. They found the most significant increase in deflection values took place in the first month after loading. In contrast, after three months of stabilisation and until the end of the test period, the deflections of the beams were almost unchanged. The nature of the curves describing the performance of the reinforced beams was similar to pure wooden beams. The deflection values were almost twice as low. The Bürgers model was used to describe the creep of all beams.

Yahyaei-Moayyed and Taheri [54] presented the results of creep tests on wooden elements reinforced with prestressed fibre composite. Power models were used to describe the rheological phenomena. The paper considered not only the rheology of wood but also of the composite reinforcement.

Recent research on the rheology of wooden beams reinforced with composite bars was presented in work by O’Ceallaigh et al. [55]. Unreinforced and reinforced glued laminated beams were subjected to long-term creep tests in a variable climate condition. A coupled hygro-mechanical finite element model was developed to predict the behaviour of FRP reinforced timber elements when stressed under long-term load and simultaneously subjected to changes in relative humidity. The model incorporates elastic, viscoelastic, mechano-sorptive, and swelling/shrinkage behaviour described using a UMAT procedure and ABAQUS FEM software.

The literature review proves that despite many studies, no scientific consensus has been reached on modelling the rheology of wood and wood-based materials and composite materials.

## 2. Materials and Methods

In order to experimentally observe the relaxation of unreinforced OSB beams and beams reinforced with composite tapes, as well as to validate the proposed theoretical model, a program of experimental research was prepared.

The elements of the experiment were single-span, simply-supported beams made of OSB boards of a high thickness (37 mm). Some of the beams were supported by attaching a reinforcement of composite CFRP tape in the expansion field. Based on the data from the manufacturers, the basic mechanical parameters of the OSB board, CFRP tape, and glue are shown in Table 1.

Five beams with no reinforcement and five reinforced beams were prepared, each with cross-section 37 × 160 mm and a total length of 2700 mm. The CFRP tape with cross-section 1.4 × 30 mm was attached in the expansion field on the whole length of the OSB beam with epoxy glue. During the bonding of the CFRP tape and later storage of the beams, all technological requirements were respected, especially with respect to moisture protection.

Before commencing the work, all beams were seasoned for six months in the laboratory, where the tests were later performed. During the seasoning and the tests, the temperature in the laboratory was maintained at 18–22 °C and the relative humidity at 40–60%.

**Figure 1:**
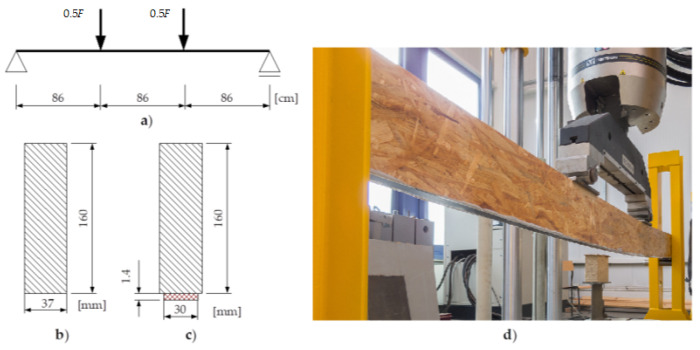
Static scheme and cross-sections of beams: (**a**) static scheme, (**b**) cross-section of OSB beam, (**c**) cross-section of OSB beam reinforced in the tensile zone with CFRP strip, (**d**) one of the beams during testing.

A four-point bending scheme according to Figure 1 was adopted. All the tests were performed with an Instron 8804 fatigue testing machine. Displacements and changes of load were measured with machine sensors.

Five bending levels were applied on the load application point, one for each of the beams of both types. The deflections were equal to 7 mm, 10.5 mm, 14 mm, 17.5 mm, and 21 mm. The last deflection corresponds to approximately 95% of the resistance to bending of a beam with no reinforcement. The deflection levels have been introduced into the beam designations. For unreinforced elements they are: OSB 7, OSB 10.5, OSB 14, OSB 17.5, and OSB 21. Whereas for reinforced beams the designations OSB CFRP 7, OSB CFRP 10.5, OSB CFRP 14, OSB CFRP 17.5, and OSB CFRP 21 were adopted. For every bending level, the test lasted 10 days. The results were recorded every minute. Before the main test, each of the beams was forced to a deflection of 14 mm. Subsequently, they were unloaded, after which the rheological tests were performed. All general instructions included in EOTA Technical Reports [56] were followed during the development of the program.

The resulting load changes in time were calculated into loss of force against the initial force ratio and averaged out separately for each of the beam types per all bending levels. Thus, an experimental relaxation curve provided as averaged relative loading changes in time was obtained for every type of beam (unreinforced and reinforced). Both curves were used to test the theoretical models described further in the article.

## 3. Results

**Figure 2:**
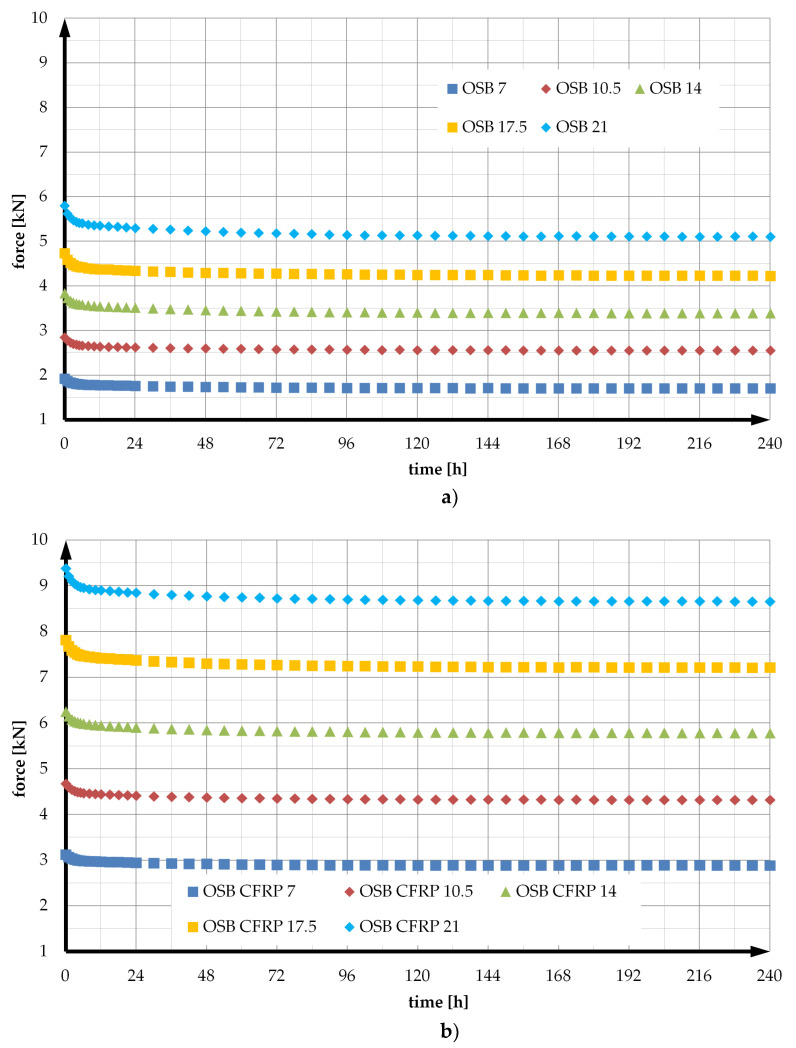
Experimental relaxation curves: (**a**) OSB beams; (**b**) OSB beams reinforced in the tension zone with CFRP tape.

The results in experimental relaxation curves at 240 h for both beam types and all deflection levels are shown in Figure 2.

The results are converted into ratios of force reduction to initial force and averaged separately for both beam types for all deflection levels. Experimental relaxation curves were obtained using averaged relative load changes in time for each beam type (OSB and OSB CFRP). Both curves were used to test the theoretical model described herein.

### 3.1. Rheological Model of OSB Beam

As shown earlier, the rheological behaviour of wood and wood-based materials is well described by multi-parameter rheological models, particularly the five-parameter model. The model is a serial combination of two Kelvin–Voigt models and a Hooke’s model. When the components are connected in series, their creep functions can be added [57]. In the face of this, the creep function of this model takes the form:

(1) $$J\left(t\right)=\frac{1}{E_{0}}+\frac{1}{E_{1}}\cdot \left(1-e^{-\frac{E_{1}\cdot t}{\eta_{1}}}\right)+\frac{1}{E_{2}}\left(1-e^{-\frac{E_{2}\cdot t}{\eta_{2}}}\right)$$

where:

*E*_0_, *E*_1_, *E*_2_*—*elasticity moduli of the rheological model elements;
*η*_1_, *η*_2_*—*viscosity moduli of the rheological model elements.

The differential form of the constitutive equation will be used to determine the relaxation function. In the assumed five-parameter model, the strain is equal to the sum of the strains of the individual components:

(2) $$\varepsilon =\varepsilon_{0}+\varepsilon_{1}+\varepsilon_{2}.$$

Furthermore, it is known that:

(3) $$\varepsilon_{0}=\frac{\sigma }{E_{0}}.$$

In the Kelvin–Voigt model, the differential form of the constitutive equation takes the form:

(4) $$\sigma =E\cdot \varepsilon +\eta \cdot D_{t}\varepsilon ,$$

where:

*D_t_*—differentiation operator.

It follows from the constitutive Equation (4):

(5) $$\sigma =E_{1}\cdot \varepsilon_{1}+\eta_{1}\cdot D_{t}\varepsilon_{1},$$

(6) $$\sigma =E_{2}\cdot \varepsilon_{2}+\eta_{2}\cdot D_{t}\varepsilon_{2}.$$

Calculating $\varepsilon_{1}$ and $\varepsilon_{2}$ from the above formulas then substituting into (2), the differential form of the constitutive equation of the five-parameter model was obtained:

(7) $$p_{0}\cdot \sigma +p_{1}\cdot D_{t}\sigma +p_{2}\cdot D_{t}^{2}\sigma =q_{0}\cdot \varepsilon +q_{1}\cdot D_{t}\varepsilon +q_{1}\cdot D_{t}^{2}\varepsilon ,$$

where:

(8) $$p_{0}=E_{0}\cdot E_{2}+E_{1}\cdot E_{2}+E_{0}\cdot E_{1},\;p_{1}=\left(E_{0}+E_{1}\right)\cdot \eta_{2}+\left(E_{2}+E_{0}\right)\cdot \eta_{1},\;p_{2}=\eta_{1}\cdot \eta_{2},\;q_{0}=E_{0}\cdot E_{1}\cdot E_{2},\;q_{1}=E_{0}\cdot \left(E_{1}\cdot \eta_{2}+E_{2}\cdot \eta_{1}\right),\;q_{2}=E_{0}\cdot \eta_{1}\cdot \eta_{2}.$$

In the case of relaxation, the deformation can be described by relation (9):

(9) $$\varepsilon \left(t\right)=\varepsilon_{0}\cdot H\left(t\right),$$

where:

*ε*_0_—strain in *t*_0_ = 0;
*H*(*t*)—Heaviside’s function.

The constitutive Equation (7) takes the form:

(10) $$p_{0}\cdot \sigma +p_{1}\cdot D_{t}\sigma +p_{2}\cdot D_{t}^{2}\sigma =q_{0}\cdot \varepsilon_{0}\cdot H\left(t\right)+q_{1}\cdot \varepsilon_{0}\cdot \delta \left(t\right)+q_{2}\cdot \varepsilon_{0}\cdot D_{t}\delta \left(t\right),$$

A Laplace transformation was performed on the compound (10):

(11) $$p_{0}\cdot \sigma +p_{1}\cdot \sigma \cdot s+p_{2}\cdot \sigma \cdot s^{2}=\frac{1}{s}\cdot q_{0}\cdot \varepsilon_{0}+q_{1}\cdot \varepsilon_{0}+q_{2}\cdot \varepsilon_{0}\cdot s.$$

From (11) the stress transformation was calculated  σ:

(12) $$\sigma =\frac{\varepsilon_{0}\cdot \left(q_{0}\cdot \frac{1}{s}+q_{1}+q_{2}\cdot s\right)}{p_{0}+p_{1}\cdot s+p_{2}\cdot s^{2}},$$

The relation (12) was transformed to the form:

(13) $$\sigma =\frac{\varepsilon_{0}\cdot \left(q_{0}\cdot \frac{1}{s}+q_{1}+q_{2}\cdot s\right)}{p_{2}\cdot \left(s-\rho_{1}\right)\cdot \left(s-\rho_{2}\right)},$$

where:

(14) $$\rho_{1}=\frac{1}{2\cdot p_{2}}\cdot \left[-p_{1}+\left(p_{1}^{2}-4\cdot p_{2}\cdot p_{0}\right){\;}^{\frac{1}{2}}\right],\;\rho_{2}=\frac{1}{2\cdot p_{2}}\cdot \left[-p_{1}-\left(p_{1}^{2}-4\cdot p_{2}\cdot p_{0}\right){\;}^{\frac{1}{2}}\right].$$

An inverse Laplace transform was performed on Equation (13):

(15) $$\sigma \left(t\right)=\frac{\varepsilon_{0}}{p_{2}\cdot \rho_{1}\cdot \rho_{2}}\cdot \{q_{0}-\frac{1}{\rho_{2}-\rho_{1}}\cdot [\rho_{2}\cdot e^{\rho_{1}\cdot t}\cdot \left(q_{0}+q_{1}\cdot \rho_{1}+q_{2}\cdot \rho_{1}^{2}\right)\;-\rho_{1}\cdot e^{\rho_{2}\cdot t}\cdot \left(q_{0}+q_{1}\cdot \rho_{2}+q_{2}\cdot \rho_{2}^{2}\right)]\}$$

Under constant deformation conditions (9), the stress during relaxation is described by the equation:

(16) $$\sigma \left(t\right)=\varepsilon_{0}\cdot E\left(t\right).$$

Considering (16), the relaxation function of the five-parameter model takes the form:

(17) $$E\left(t\right)=\frac{1}{p_{2}\cdot \rho_{1}\cdot \rho_{2}}\cdot \{q_{0}-\frac{1}{\rho_{2}-\rho_{1}}\cdot [\rho_{2}\cdot e^{\rho_{1}\cdot t}\cdot \left(q_{0}+q_{1}\cdot \rho_{1}+q_{2}\cdot \rho_{1}^{2}\right)\;-\rho_{1}\cdot e^{\rho_{2}\cdot t}\cdot \left(q_{0}+q_{1}\cdot \rho_{2}+q_{2}\cdot \rho_{2}^{2}\right)]\}$$

Using Equation (16) and knowing the stresses at time t and t=0:

(18) $$\sigma \left(t\right)=\frac{M\left(t\right)}{W}\;\mathrm{and}\;\sigma_{0}=\frac{M_{0}}{W},$$

and:

(19) $$\varepsilon_{0}=\frac{\sigma_{0}}{E_{0}}.$$

where:

M(t) and $M_{0}$—bending moment in t and t=0;

W—bending strength indicator.

We have:

(20) $$M\left(t\right)=M_{0}\cdot \frac{E\left(t\right)}{E_{0}},$$

hence

(21) $$F\left(t\right)=F_{0}\cdot \frac{E\left(t\right)}{E_{0}}.$$

**Figure 3:**
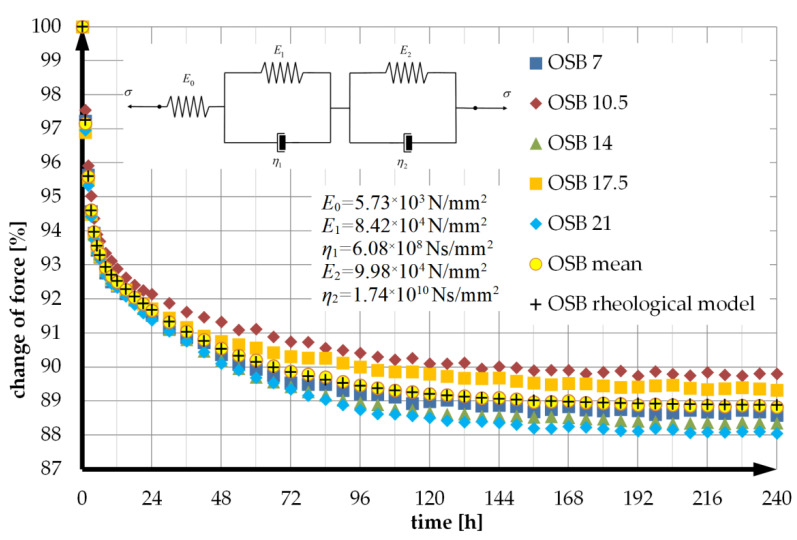
Experimental OSB beam relaxation functions, mean function, and theoretical model with parameter values.

The values of the unknowns *E*_0_, *E*_1_, *E*_2_, *η*_1_, *η*_2_ were calculated using Formula (21), the experimental values of the force *F*(*t*) shown in Figure 2a, and the optimisation procedures available in the spreadsheet. Plots of the average experimental relaxation function and the theoretical model and its parameter values and scheme are shown in Figure 3.

The average ratios of the force values in successive measurements to the force value *F*_0_ at time *t* = 0 from all deflection levels (curve labeled OSB mean in Figure 3) were used for the calculations.

Wood and wood-based materials are non-homogeneous so difficulties can be expected in obtaining high reproducibility of results. This problem is particularly pronounced for wood due to numerous defects that naturally occur in this material. Wood-based materials, including OSB, are free from this problem. In addition, the beams for this study were cut from one large sheet of thick OSB, thus ensuring virtually identical material parameters for each sample. As a result, there are only small differences in results for particular deflection levels, considered for ratios of force at time t to initial force at time *t* = 0. A useful tool for evaluating the scatter of results is the percentage relative standard deviation %RSD, defined as the quotient of the standard deviation SD and the mean value of the force at each time of measurement:

(22) $$\%RSD\left(F_{e}\left(t_{k}\right)\right)=\frac{SD\left(F_{e}\left(t_{k}\right)\right)}{\bar{F_{e}\left(t_{k}\right)}}\cdot 100\%,$$

where:

$\bar{F_{e}\left(t_{k}\right)}$—average force from the experiment at time $t_{k}$

Values $\%RSD\left(F_{e}\left(t_{k}\right)\right)$ was calculated for each time t_k_ and then averaged. For unreinforced OSB beams, this value is 0.61%.

Based on the results obtained, it was found that the five-parameter model describes the relaxation of OSB beams very well. The mean percent relative square deviation expressed by the formula was used as a measure of the fit of the theoretical calculation results to the experimental data:

(23) $$s=\sqrt{\frac{1}{n}\sum_{k=1}^{n}{\left(\frac{F_{t}\left(t_{k}\right)-\bar{F_{e}\left(t_{k}\right)}}{F_{t}\left(t_{k}\right)}\right)}^{2}}\cdot 100\%,$$

where:

$F_{t}\left(t_{k}\right)$—force from the theoretical model at time $t_{k}$;
*n*—number of force measurements.

Due to the tests at different load levels, calculations were performed not for the values of the forces but for their ratios to the corresponding initial force at time *t* = 0.

The smaller the deviation *s* the better the fit of the theoretical model to the experimental data and this value was minimized in the optimisation calculations used to determine the values of the model constants. For the applied five-parameter rheological model the deviation value was *s* = 0.037%.

### 3.2. Rheological Model of OSB Beam with CFRP Reinforcement

The rheological behaviour of wood and wood-based materials is well described by multi-parameter rheological models, particularly the five-parameter model. Experimental studies and theoretical analyses using these models show the significant influence of rheological phenomena on the wooden and wood-based structures behaviour. The rheological properties of a single-material beam are expressed by increasing deformations (creep) or decreasing stress values (relaxation) during exploitation. Different rheological properties of the materials used in multi-material beams additionally affect the redistribution of stresses in cross-sections, even if the global cross-sectional forces are constant.

The negative effects of rheological phenomena can be reduced by using CFRP reinforcements. The wooden structure and glued CFRP strips create a layered system. The theoretical basis of a layered structure behaviour can be found in work [58]. The following assumptions were established:

1. A cross-section of a beam is symmetrical about the vertical axis;
2. A beam is composed of layers made of the linear elastic (CFRP strip) and viscoelastic materials (OSB);
3. The cross-sections remain plane;
4. The layers are perfectly joined using a very thin glue layer;
5. The influence of the glue layer is neglected.

In the elastic range, the assumptions noted above lead to a classical solution where the properties of cross-section are calculated based on the Young modulus ratio of the materials used.

The deformation under external load is determined by a geometrical relation in any fibre of the *i*th layer of the beam:

(24) $$\varepsilon^{i}=Κ\cdot z^{i},$$

where:

*K*—curvature;
*z^i^—*distance of the considered fibre from the neutral axis;

which after differentiation takes the form:

(25) $$d\varepsilon^{i}=dΚ\cdot z^{i}.$$

The static equilibrium equation of normal forces *N* in each layer is equal to 0:

(26) $$N=\sum_{i=1}^{n}N^{i}=0.$$

*N^i^* is a result of normal stresses *σ^i^* in ith layer with cross-section *A^i^*:

(27) $$N^{i}=\int_{A^{i}}^{}\sigma^{i}{\mathrm{dA}}^{i}.$$

The rheological behaviour of the material of each inclusion is described by the integral equation of a linear viscoelastic medium:

(28) σ=E∗dε.

where:

*E*—material relaxation function;

∗—convolution product.

By substituting the relation (28) into (27) we obtain:

(29) $$N^{i}=\int_{A^{i}}^{}E^{i}*d\varepsilon^{i}{\mathrm{dA}}^{i},$$

given the geometric relation (25):

(30) $$N^{i}=\underset{A^{i}}{\int }E^{i}*dΚ\cdot z^{i}{\mathrm{dA}}^{i}.$$

In view of this, the relation (30) takes the form:

(31) $$N^{i}=\sum_{i=1}^{n}\int_{A^{i}}^{}E^{i}*dΚ\cdot z^{i}{\mathrm{dA}}^{i}=0,$$

and after conversion:

(32) $$N=\sum_{i=1}^{n}E^{i}*dΚ\cdot \int_{A^{i}}^{}z^{i}{\mathrm{dA}}^{i}=0.$$

The integral in Equation (32) is the static moment of the layer field $A^{i}$ relative to the neutral axis:

(33) $$S^{i}=\int_{A^{i}}^{}z^{i}{\mathrm{dA}}^{i},$$

so Equation (32) can be written in the form:

(34) $$N=\sum_{i=1}^{n}E^{i}*dΚ\cdot S^{i}=0.$$

It is resulting:

(35) $$\sum_{i=1}^{n}E^{i}\cdot S^{i}=0.$$

The time depending position of the neutral axis could be determined by the Equation (35). The bending moment in the *i*th inclusion can be calculated from the formula:

(36) $$M^{i}=\int_{A^{i}}^{}\sigma^{i}\cdot z^{i}{\mathrm{dA}}^{i},$$

considering the geometrical relation (25) and the integral equation of a linearly viscoelastic medium (28):

(37) $$M^{i}=\int_{A^{i}}^{}E^{i}*dΚ\cdot {\left(z^{i}\right)}^{2}\;{\mathrm{dA}}^{i}.$$

The above equation can be transformed to the form:

(38) $$M^{i}=E^{i}*dΚ\cdot \int_{A^{i}}^{}{\left(z^{i}\right)}^{2}\;{\mathrm{dA}}^{i},$$

where:

(39) $$I^{i}=\int_{A^{i}}^{}{\left(z^{i}\right)}^{2}\;{\mathrm{dA}}^{i},$$

is the moment of inertia of the field $A^{i}$ relative to the neutral axis. After considering (39) Equation (38) becomes

(40) $$M^{i}=E^{i}*dΚ\cdot I^{i}.$$

The total bending moment in the cross-section is equal to the sum of the partial bending moments in each layer:

(41) $$M=\sum_{i=1}^{n}M^{i}=\sum_{i=1}^{n}E^{i}*dΚ\cdot I^{i}.$$

From Equation (40), the convolution product can be determined:

(42) $$E^{i}*dΚ=\frac{M^{i}}{I^{i}}.$$

The same product can be determined from Formulas (25) and (28):

(43) $$E^{i}*\mathrm{dK}=\frac{\sigma^{i}}{z^{i}}.$$

After comparing the right-hand sides of Equations (42) and (43), the formula describing the normal stress distribution in the ith layer was obtained:

(44) $$\sigma^{i}=\frac{M^{i}\cdot z^{i}}{I^{i}}.$$

This formula is analogous to the classical relation for the distribution of normal stresses in a homogeneous bending beam. However, the moment is still an unknown quantity. It can be determined using the strain compatibility condition. Equations (40) and (41) give the relations:

(45) $$dΚ=M^{i}*{\left(E^{i}\cdot I^{i}\right)}^{-1},$$

(46) $$dK=M*{\left(\sum_{i=1}^{n}E^{i}\cdot I^{i}\right)}^{-1}.$$

By comparing the right-hand sides of the above formulae, it is possible to derive a relation describing the bending moment in the ith layer:

(47) $$M^{i}=E^{i}\cdot I^{i}*{\left(\sum_{i=1}^{n}E^{i}\cdot I^{i}\right)}^{-1}*M.$$

Unfortunately, the convolution product occurring in the above relations is the cause of great difficulty in the exact solution of these equations. Therefore, an approximate method is used, which consists of replacing the convolution product by a sum with a variable summation limit [58]. In this case, Equation (47), defining the total bending moment in the cross-section, takes the form:

(48) $$M\left(t_{m}\right)=\sum_{j=0}^{m}\{[I^{i}\left(t_{m}\right)\cdot E^{i}\left(t_{m}-t_{j}\right)]\cdot \Delta Κ\left(t_{j}\right)\}.$$

**Figure 4:**
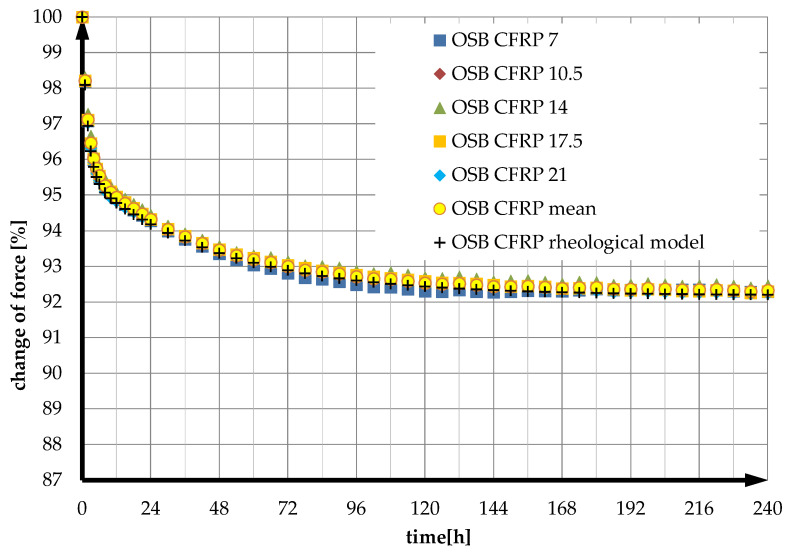
Experimental relaxation functions of OSB beams reinforced with CFRP tape, mean function, and theoretical model.

The values of the time-decreasing bending moment *M*(*t_m_*) were calculated using Formula (48). Graphs of the experimentally averaged relaxation function and the theoretical model are shown in Figure 4. As in the case of unreinforced beams, the deflection values $\%RSD\left(F_{e}\left(t_{k}\right)\right)$ were calculated for each instant *t_k_* according to Equation (22) and then averaged. For the reinforced OSB beams, this value is 0.11%.

Based on the results obtained, it was found that the presented rheological model of an OSB beam reinforced with CFRP tape describes the relaxation of such beams very well. The squared deviation calculated according to Formula (23) is in this case *s* = 0.15%.

The values of geometrical characteristics of both cross-sections (OSB only and OSB + CFRP) were obtained using Equation (33), which allowed determining the stiffness of the strengthened beam. The stiffness occurred 63% higher than the OSB beam without reinforcement.

### 3.3. Redistribution of Stresses in the Cross-Section of the OSB Beam with CFRP Reinforcement

Using the relations presented in the previous section, especially (44), normal stress values were calculated. The importance of normal stresses at the top (compression) and bottom (tension) edges of the OSB beam and CFRP strip are shown in Table 2. The presented extreme stress values were calculated under deflection of the reinforced beam equal to 21 mm in time 0 h and 240 h.

**Figure 5:**
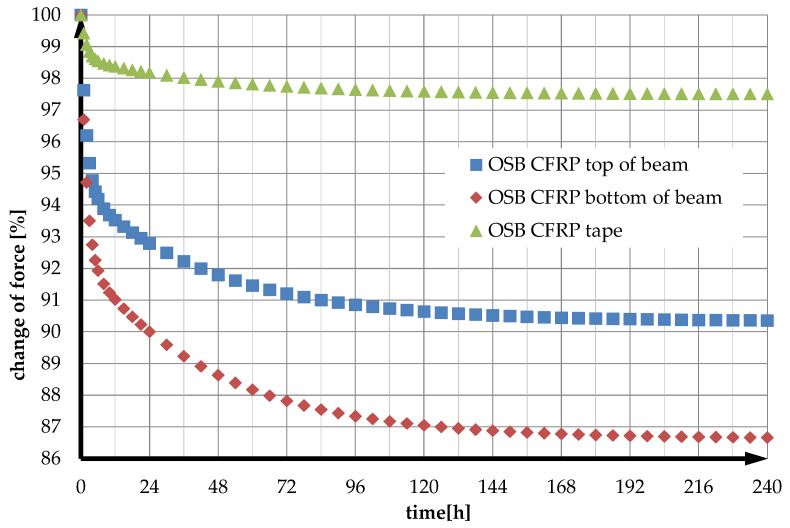
Stress redistribution and relaxation in the beam and the reinforcing strip’s tensile (bottom) and compressive (top) zones.

The values of relative stress changes in time were then calculated by dividing the stress values at a given moment by the stress at time *t* = 0. Calculations were performed for both the top (compression) and bottom (tension) edges of the reinforced OSB beam and the CFRP reinforcing strip. The results are presented in Figure 5.

The analysis of the results concludes that in the OSB beams with glued composite reinforcement under bending, a significant redistribution of stresses within the cross-section occurs. The most negligible stress reduction occurs in the CFRP strip (97.51% of stresses at time *t* = 0). The stresses at the top edge of the beam decrease to a relatively small extent (90.36% of stresses at time *t* = 0). In contrast, stresses at the lower edge of the OSB beam decrease to a greater extent (86.67% of stresses at time *t* = 0). In summary, stresses decrease in each part of the cross-section but at different rates.

## 4. Discussion

There is no one generally accepted theoretical model of the performance of a wooden beam with glued-in reinforcement under both short- and long-term loads. In the latter case, as a result of different rheological properties of the materials (wood, glue, reinforcing composite inserts), stress redistribution in the beam will occur. This problem has also not been fully resolved to date. A key problem is the determination of the adhesive bond vulnerability. Its existence and nature or possible lack determines the choice of theory describing the work of the reinforced element. According to Jasieńko [52], high susceptibility of adhesive bond affects the reduction of stiffness of the whole element and redistribution of stresses in the composite cross-section. However, for the joints made with low deformability adhesives, with relative elongation at break up to approximately 5%, full cooperation of the wooden beam with the reinforcing bars can be assumed.

Considering the above remarks, theoretical studies on the cooperation of wood, adhesive layer, and reinforcing materials can be divided into three groups depending on the adopted scheme of internal structure of the reinforced element. The first group includes solutions based on the concept of equivalent cross-section created by importing geometric characteristics of glued reinforcement to the cross-section of wood. In this method it is assumed that the adhesive layer is thin and shows the same physical properties as wood and is not susceptible.

The second group of the solutions is based on the susceptibility of the adhesive bond. It is generally assumed that the shear stress in the adhesive layer is directly proportional to the linear displacement of reinforcement and timber.

The third group of solutions is based on the theory of sandwich construction. An extensive discussion of various aspects of this approach including rheological phenomena was presented by Kubik [58].

In the author’s opinion, this approach is one of the most appropriate and it has been used here. The adoption of the sandwich model solves two problems: accounting for the rheological properties of all the materials used and the susceptibility of the adhesive joint. This paper presents small scale model tests. In cooperation with a manufacturer of OSB and I-beams of wood and wood-based materials, the authors plan to carry out tests on natural scale elements in order to clearly determine the possibility of replacing I-beams with beams made of thick OSB. The planned research will also include the use of composite reinforcements that, in contrast to the model tests presented here, will be optimally selected considering the load level and economic considerations. In the model tests presented here, due to the huge difference in strength between OSB and composite tape and the low load on the tape and adhesive bond, an assumption of elastic work of these elements was made without considering their rheology. However, this assumption had no effect on the presented equations describing the work of sandwich beams. It only simplified calculations for this particular case. In contrast, in the planned natural scale tests with optimised reinforcement carrying higher loads, it will not be appropriate to ignore the influence of the rheology of the reinforcing materials and the susceptibility of the grout. At this point, a great advantage of the proposed mathematical model based on the theory of sandwich structures can be seen—the derived equations allow for including in the calculations any number of layers of linear visco-elastic materials. This makes it possible to consider the rheological properties of the reinforcing tape and the adhesive. Furthermore, in the case of an adhesive bond, the theory of sandwich structures allows two approaches. With a relatively thick weld, it can be treated as another reinforcing layer. In contrast, in the case of a susceptible thin weld, the solution presented in Kubik’s work [58] for the layered bar with layer slips can be used. Of course, in addition to its advantages and versatility, the presented solution also has the disadvantage of tedious calculations of the convolution product (Equations (47) and (48)). This disadvantage can be minimized by numerical methods.

## 5. Conclusions

The experimental investigations and theoretical calculations allowed us to draw the following conclusions:

1. An excellent way to increase the load capacity of wood-based structures and diminish the influence of the wood’s defects is to place a composite reinforcement in the tension zone of the beam;
2. The five-parameter model describes the rheology of OSB very well;
3. The proposed theoretical model of reinforced beam displays good agreement with experimental data;
4. A significant redistribution of stresses in the cross-section occurs in the reinforced elements;
5. The reinforced beams show a higher stiffness of approximately 63% and carry proportionally higher loads than unreinforced beams at the same deflection values.

## Figures and Tables

**Table 1:** Mechanical parameters of used materials (manufacturers’ data).

Material/Parameter	OSB	CFRP	Epoxy Glue
cross-section [mm]	37 × 160	1.4 × 30	0.5 × 30
length [mm]	2700	2700	2700
bending strength [N/mm^2^]	18.9	–	–
tensile strength [N/mm^2^]	0.30	3.50 × 10^3^	26
Young’s modulus [N/mm^2^]	5.74 × 10^3^	2.10 × 10^8^	1.12 × 10^7^

**Table 2:** Normal stress [N/mm^2^] in beam OSB CFRP under deflection 21 mm.

Time	OSB Top	OSB Bottom	CFRP
0 [h]	18.87	12.64	463.25
240 [h]	17.05	10.95	451.72
